# Alcohol consumption and breast cancer prognosis after breast cancer diagnosis: a systematic review and meta‑analysis of the Japanese Breast Cancer Society Clinical Practice Guideline, 2022 edition

**DOI:** 10.1007/s12282-023-01455-4

**Published:** 2023-04-08

**Authors:** Tsunehisa Nomura, Masaaki Kawai, Yuna Fukuma, Yoshikazu Koike, Shinji Ozaki, Motoki Iwasaki, Seiichiro Yamamoto, Kiyoshi Takamatsu, Hitoshi Okamura, Masami Arai, Shoichiro Ootani, Hiroji Iwata, Shigehira Saji

**Affiliations:** 1 Department of Breast and Thyroid Surgery, Kawasaki Medical School, 577 Matsushima, Kurashiki, 701-0192 Japan; 2 Department of Surgery I, Yamagata University Hospital, Yamagata, Japan; 3 Department of Breast Surgery, Hiroshima Prefectural Hospital, Hiroshima, Japan; 4 Division of Epidemiology, National Cancer Center Institute for Cancer Control, Tokyo, Japan; 5 Shizuoka Graduate University of Public Health, Shizuoka, Japan; 6 Department of Obstetrics and Gynecology, Tokyo Dental College Ichikawa General Hospital, Ichikawa, Japan; 7 Department of Psychosocial Rehabilitation, Graduate School of Biomedical and Health Sciences, Hiroshima University, Higashihiroshima, Japan; 8 Department of Clinical Genetics, Juntendo University, Graduate School of Medicine, Tokyo, Japan; 9 Department of Ohtani Shoichiro Breast Clinic, Hiroshima, Japan; 10 Department of Breast Oncology, Aichi Cancer Center Hospital, Nagoya, Japan; 11 Department of Medical Oncology, Fukushima Medical University, Fukushima, Japan

**Keywords:** Alcohol consumption, Breast cancer, Systematic review, Meta-analysis

## Abstract

Alcohol consumption is internationally recognized as one of the compelling risk factors for breast cancer, but it does not necessarily correlate with the prognosis of breast cancer patients. Alcohol consumption in breast cancer patients was addressed in the 2022 Breast Cancer Clinical Practice Guidelines. A systematic review and meta-analysis of epidemiological studies on alcohol consumption and breast cancer recurrence, breast cancer-related mortality, all-cause mortality, and cardiovascular disease mortality in breast cancer patients was performed. The PubMed, Cochrane Library, and Ichushi-Web databases were searched for relevant publications reporting cohort or case–control studies published until March 2021. A total of 33 studies (32 cohort studies and 1 case–control study) met the eligibility criteria; 4638 cases of recurrence, 12,209 cases of breast cancer-specific mortality, and 21,945 cases of all-cause mortality were observed. With regard to breast cancer recurrence, 7 studies assessed pre-diagnosis alcohol consumption (relative risk (RR) 1.02, 95% confidence interval (95% CI) 0.77–1.37, p = 0.88) and 3 studies assessed post-diagnosis alcohol consumption (RR 0.96, 95% CI 0.85–1.10, p = 0.57), and no significant increase or decrease in risk was observed. With regard to breast cancer-related mortality, 19 studies assessed pre-diagnosis alcohol consumption (RR 1.02, 95% CI 0.93–1.11, p = 0.69), 9 studies assessed post-diagnosis alcohol consumption (RR 0.96, 95% CI 0.77–1.19, p = 0.70), and no significant increase or decrease in risk was observed. With regard to all-cause mortality, 18 studies assessed pre-diagnosis alcohol consumption (RR 0.90, 95% CI 0.82–0.99, p = 0.02), 8 studies assessed post-diagnosis alcohol consumption (RR 0.88, 95% CI 0.74–1.02, p = 0.08), and pre-diagnosis alcohol consumption was associated with a significantly decreased risk. With regard to cardiovascular disease mortality and alcohol consumption, 2 studies assessed it, and the RRwas 0.47 (95% CI 0.28–0.79, p = 0.005), showing that alcohol consumption was associated with a significantly decreased risk. The limitations of this study are that drinking status was mainly based on a questionnaire survey, which is somewhat inaccurate and has many confounding factors, and the cut-off value for the maximum alcohol intake in many studies was low, and it is possible that the actual intake was only an appropriate amount. In many countries, a standard drinking amount is set, and wise decisions are required.

## Introduction

Alcohol consumption is internationally recognized as one of the compelling risk factors for breast cancer. Ethanol contained in alcoholic beverages and its metabolite acetaldehyde have been experimentally shown to be carcinogenic [1]. However, the increased risk of breast cancer due to alcohol consumption does not necessarily correlate with the prognosis of breast cancer patients. Even in breast cancer patients, there are many occasions where alcoholic beverages are preferred in daily life or social gatherings. It is a modifiable factor through individual effort, and accurate information is required.

In June 2022, the Japanese Breast Cancer Society practice guidelines, the 2022 edition, was published by the Japanese Breast Cancer Society. In the guidelines, we addressed the clinical question, “Is alcohol consumption associated with prognosis in breast cancer patients?” To make recommendations for this clinical question, we assessed the benefits and harms of alcohol consumption after patients were diagnosed with breast cancer by performing a systematic review and meta-analysis of the relevant literature. The results of this systematic review and meta-analysis are reported.

## Methods

A quantitative and qualitative systematic review was performed according to the Minds Handbook for Clinical Practice Guideline Development Ver. 3.0, 2020 [2]. Regarding alcohol consumption in breast cancer patients, the importance of outcomes was examined based on the Minds Handbook. Breast cancer recurrence, breast cancer mortality, and all-cause mortality were judged to be important as harms, and the cardiovascular disease reduction effect was judged to be important as a benefit.

### Search strategy and selection criteria

The PubMed, Cochrane Library, and Ichushi-Web databases were searched using the terms “breast neoplasms”, “breast cancer”, “alcohol drinking”, “alcohol consumption”, “prognosis”, “recurrence”, “survival”, and “mortality”. The search was limited to articles published up to March 2021.

According to these criteria, two authors (TN and YK) independently reviewed all titles and abstracts of the retrieved articles. The full articles were evaluated for relevance. Systematic reviews were eligible when they were judged to be of moderate quality according to AMSTAR [3] and included studies fulfilling the inclusion criteria. Authors were allowed to independently search the articles and add them to the list when they met all inclusion criteria (hand-searching). Reviews, articles on breast cancer incidence, very low population numbers, and articles with missing outcome data were excluded.

### Quality assessment and data extraction

The qualities of the trials selected through the process described above were evaluated according to the Minds Handbook for Clinical Practice Guideline Development Ver. 3.0, 2020 [2].

### Statistical analysis

Review Manager software 5.4 (https://review-manager.software.informer.com/5.4/) was used for statistical analysis. The summary of relative risk (RR) (hazard ratio or risk ratio) and 95% confidence intervals (95% CI) for the highest and lowest categories of alcohol intake were estimated using the inverse variance method.

RR < 1 favored alcohol intake. The heterogeneity of the trials’ results was assessed by inspecting graphical presentations; the Chi-squared test was used to evaluate heterogeneity, and I^2^ statistics were used to evaluate inconsistency. Significant heterogeneity was defined as I^2^ statistic > 50%. All analyses were performed using a random effects model. A funnel plot estimating the precision of a trial was examined for asymmetry to estimate publication bias. The outcomes of this meta-analysis were breast cancer recurrence, breast cancer-specific death, all-cause and cardiovascular disease mortality.

## Results

### Literature search results

**Fig. 1:**
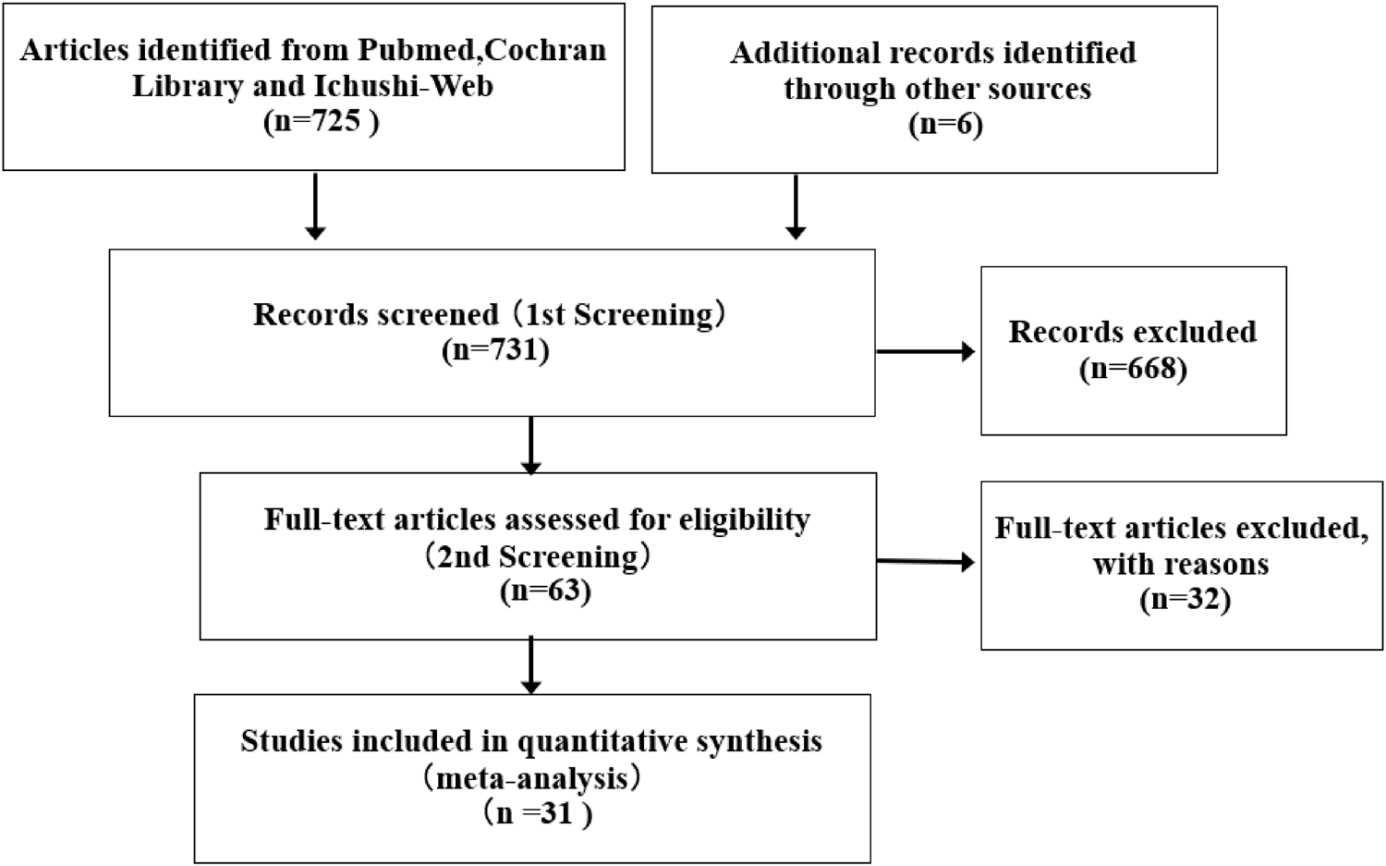
Results of the search

The database search identified 725 potentially relevant articles, and hand-searching was used to identify six studies. In the 1st screening, 668 studies were excluded, leaving 63 systematic review articles. In the 2nd screening, 31 papers met the criteria and were judged eligible (Fig. 1). Alcohol consumption and breast cancer recurrence, breast cancer-related morality, all-cause mortality in breast cancer patients and cardiovascular disease mortality in breast cancer patients or healthy participants were assessed.

### Overview of included studies

Table 1 presents an overview of the characteristics of the included studies of pre- and post-diagnosis alcohol consumption and/or breast cancer recurrence, breast cancer-related mortality, all-cause mortality, and cardiovascular disease mortality. All studies meeting the inclusion criteria were observational, with 32 being cohort studies and 1 a case–control study. Data collection ranged between 1982 and 2013. Effect measures used were the hazard ratio or the risk ratio. The studies included in this systematic review presented data from 4638 cases of recurrence, 12,209 cases of breast cancer-related mortality, 21,945 cases of all-cause mortality, and 7727 cases of cardiovascular disease mortality. The participants were women diagnosed with in situ or invasive breast cancer (TNM Stage 0-IC). Median or mean follow-up was 31.56 months to 11.3 years. Exposure times were pre-diagnosis 24, post-diagnosis 7, and pre- and post-diagnosis 2. Alcohol exposure varied from over 4 g/day to over 30 g/day.

**Table 1:** Characteristics of included studies of pre- and post-diagnosis alcohol consumption and/or breast cancer recurrence, mortality (breast cancer, all-cause, cardiovascular disease)

References	Study	Study design	Deta collection period	Effect measure	Sample size (*N*)	Recurrence	Breast deaths	All-cause deaths	Cardiovascular deaths	Follow up (median or mean)	Exposure time (pre/post diagnosis)	Stage	Exposure
[4]	Hebert 1998	Cohort	1982–1984	RR	472	109	73	87	–	8–10 y	Pre	I–IIIa	12 Oz beer
[5]	Saxe 1999	Cohort	1989–1991	HR	149	28	26	–	–	5 y <	Pre	All	2drink/d <
[6]	Muscat2003	Case–control	1994–1996	RR	224	30	–	–	–	3.6 y	Pre	All	1drink/d <
[7]	Brewster2007	Cohort	1985–2000	HR	2327	332	–	–	–	5 y <	Pre	I–II	heavy
[8]	Vrieling2012	Cohort	2001–2005	HR	2522	247	235	316	20	4.4–7.4 y	Pre	I–IV	12 g <
[9]	Holm2013	Cohort	1993–1997	HR	1052	110	106	–	–	6 y	Pre	I–IIIa	20 g <
[10]	Kowalski2018	Cohort	2007–2012	HR	1399	358	–	–	–	31.56 m (mean)	Pre	I–IV	7drink/w <
[11]	Flatt2010	Cohort	1995–2000	HR	3088	518	262	315	–	7.3 y	Pre	I–IIIa	300 g/m <
[12]	Kwan2013	Cohort	1990–2006	HR	9329	1487	911	1542	156	10.3 y	Post	I–IIIa	6 g/d <
[13]	Nechuta2016	Cohort	1990–2006	HR	6596	1309	–	1427	–	10.5 y	Post	I–III	12 g/d <
[14]	Lowry2016	Cohort	No info	HR	7835	–	622	1625	–	7.9 y	Pre/post	0–III	7drink/w <
[15]	Hellmann2010	Cohort	1976–1978, 1981–1983, 1991–1994, 2001–2003	HR	528	–	178	323	–	7.8 y	Pre	Local-metastases	14unit/w <
[16]	Newcomb2013	Cohort	1988–1995, 1997–2008	HR	22,890	–	3484	7780	1531	11.3 y	Pre/post	All	10drink/w <
[17]	Din2016	Cohort	1984–1985,1987–1988	HR	939	–	303	–	–	11 y	Pre	All	10-36drink/w <
[18]	Weaver2013	Cohort	1996–2001	HR	1097	–	92	154	–	86 m (mean)	Post	All	4drink/d <
[19]	Ali(pooled)												
	SEARCH	Cohort	1991–1996	HR	8446	–	765	945	–	6 y	Post	I–IV	14drink/w <
	EPIC	Cohort	1992–2000	HR	10,561	–	174	395	–	7 y	Pre	I–IV	14drink/w <
	BCAC	Cohort	No information	HR	10,232	–	355	963	–	6 y	Pre	I–IV	14drink/w <
[20]	Harris2012	Cohort	1987–2008	HR	3146	–	385	860	–	8.2 y	Pre	I–IV	10 g/d <
[21]	Dal Maso2008	Cohort	1991–1994	HR	1453	–	398	503	–	12.4 y	Pre	I–IV	2drink/d <
[22]	Borugian2004	Cohort	1991–1992	RR	603	–	112	–	–	8 y	Pre	0–III	1% increases in energy
[23]	Allemani2011	Cohort	1987–1992	RR	264	–	43	–	–	7 y	Pre	I–IV	13 g/d <
[24]	Jain2000	Cohort	1980–1985	HR	58,926	–	223	–	–	10.3 y	Pre	No info	20 g/d <
[25]	Thun1997	Cohort	1982–1991	RR	251,420	–	691	–	5517	9 y	Pre	All	4drink/d <
[26]	Ma2019	Cohort	1994–1998	HR	4523	–	824	1055	–	8.6 y	Pre	No info	7drink/w <
[27]	Minami2019	Cohort	1997–2013	HR	1420	–	193	261	–	8.6 y	Pre	All	ever/current
[28]	Zeinomar2017	Cohort	1996–2011	HR	1116	–	58	211	–	9.1 y	Pre	All	3drink/w <
[29]	Beasley2011	Cohort	1998–2001	HR	4441	–	137	525	–	5.5 y	Post	Local and regional	quintile 5(med 15%kcal)
[30]	Fuchs1995	Cohort	1980, 1984, 1986	RR	85,709	–	350	2658	503	12 y	Post	All	30 g/d <
[31]	Breslow2010	Cohort	1988–1991,1997–2004	RR	184,764	–	677	–	–	11 y	Post	All	7drink/w <
[32]	Rohan1993	Cohort	1982–1984	HR	412	–	112	–	–	5.5 y	Pre	No info	10 g/d <
[33]	Reding2008	Cohort	1983–1992	HR	1286	–	364	–	–	12 y	Pre	All	7drink/w <
[34]	Zhang1995	Cohort	1986–1991	RR	698	–	56	–	–	2.9 y	Pre	All	4 g/d <

HR hazard ratio, RR risk ratio

### Evaluation of the risk of bias

The Cochrane risk-of-bias assessment was performed to evaluate the quality of the 33 included studies (Table 2). Non-exposed cohort bias was present in all studies because these studies were observational and not randomly assigned. On the other hand, there was a low risk of allocation concealment, blinding of participants and personnel, and selective reporting. Incomplete outcome data were rated as unclear risk of bias in 6 cases with an observation period of less than 5 years and as high risk of bias in 1 case with an observation period of less than 3 years. Regarding the adjustment of confounding factors item, there were 7 studies in which adjustment for confounding factors was hardly performed, and 5 studies in which it was evaluated as inadequate.

**Table 2:**
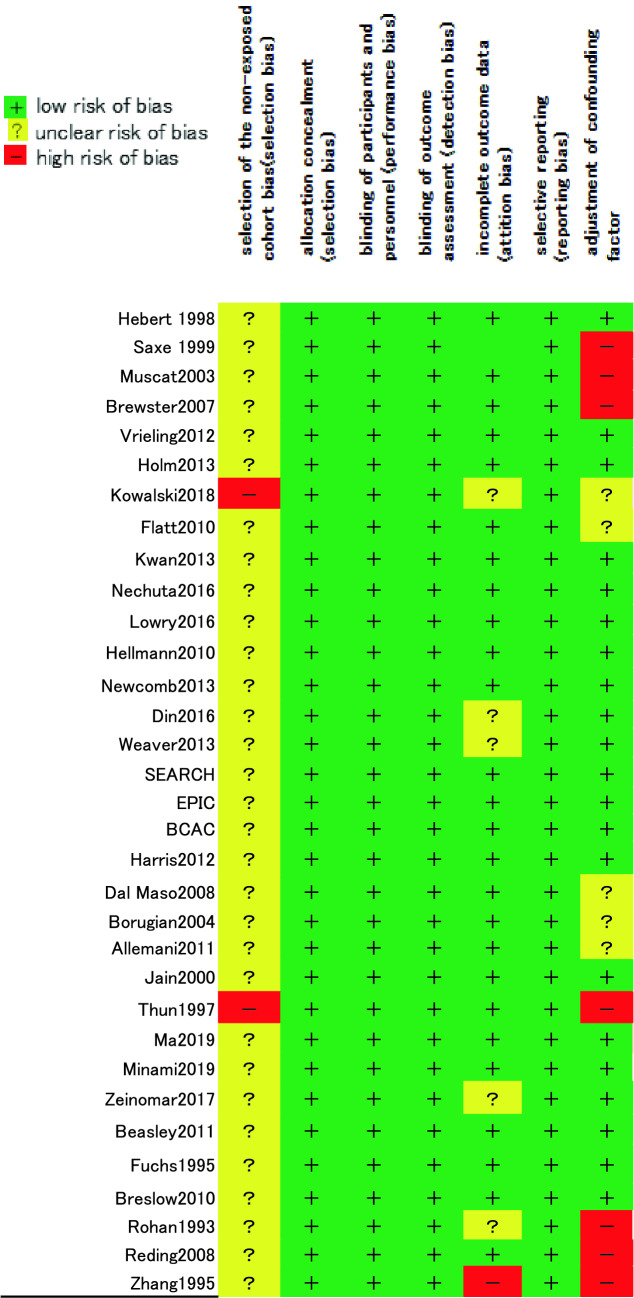
Risk of bias summary

### Association between alcohol consumption and breast cancer recurrence

There were 7 pre-diagnosis studies [4–10] and 3 post-diagnosis studies [11–13] of alcohol consumption and breast cancer recurrence in breast cancer patients. The risk of recurrence increased in two pre-diagnosis studies [4, 9] and in one post-diagnosis study limited to postmenopausal cases [12], but the risk was not significant in other studies.

**Fig. 2:**
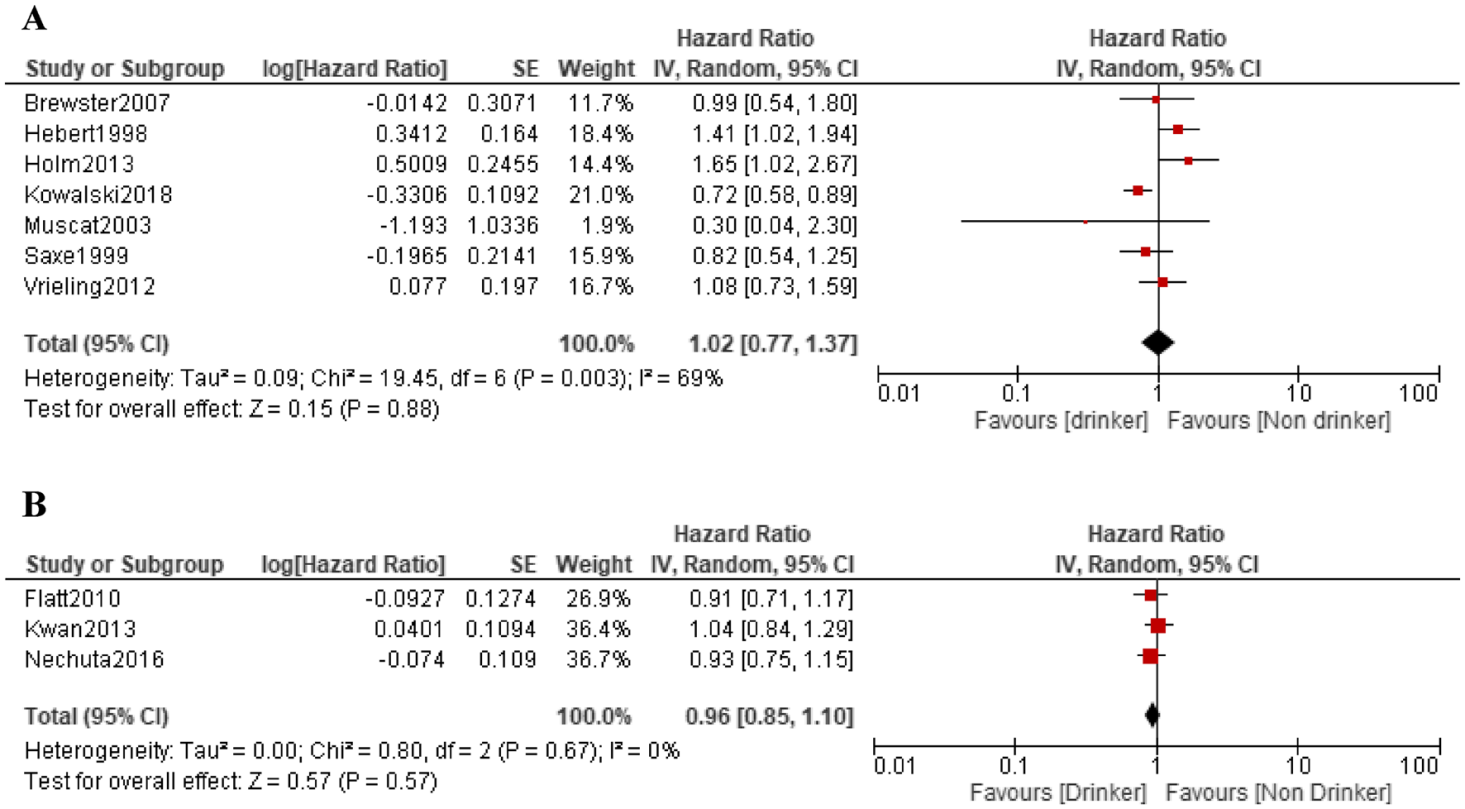
Forest plots of the hazard ratio for alcohol intake and breast cancer recurrence. **A** Pre-diagnosis; **B** post-diagnosis

In the meta-analysis of seven studies, the RR for pre-diagnosis alcohol intake was 1.02 (95% CI 0.77–1.37, p = 0.88), showing high heterogeneity in the effect (I^2^ = 69%), and no significant risk was observed. The RR for the post-diagnosis alcohol intake was 0.96 (95% CI 0.85–1.10, p = 0.57), showing no heterogeneity in the effect (I^2^ = 0%), and no significant risk was observed (Fig. 2A, B).

### Association between alcohol consumption and breast cancer-related mortality

There were 21 pre-diagnosis studies [4, 8, 9, 14–28] and 9 post-diagnosis studies [12, 14, 16, 19, 29–32] of alcohol consumption and breast cancer-related mortality in breast cancer patients. For pre-diagnosis alcohol intake, increased risk was observed in four studies [4, 8, 23, 24] and decreased risk in two studies [26, 27], but the risk was not significant in other studies. For post-diagnosis alcohol intake, increased risk was observed in 1 study [30] and 1 study [12] when limited to postmenopausal cases.

**Fig. 3:**
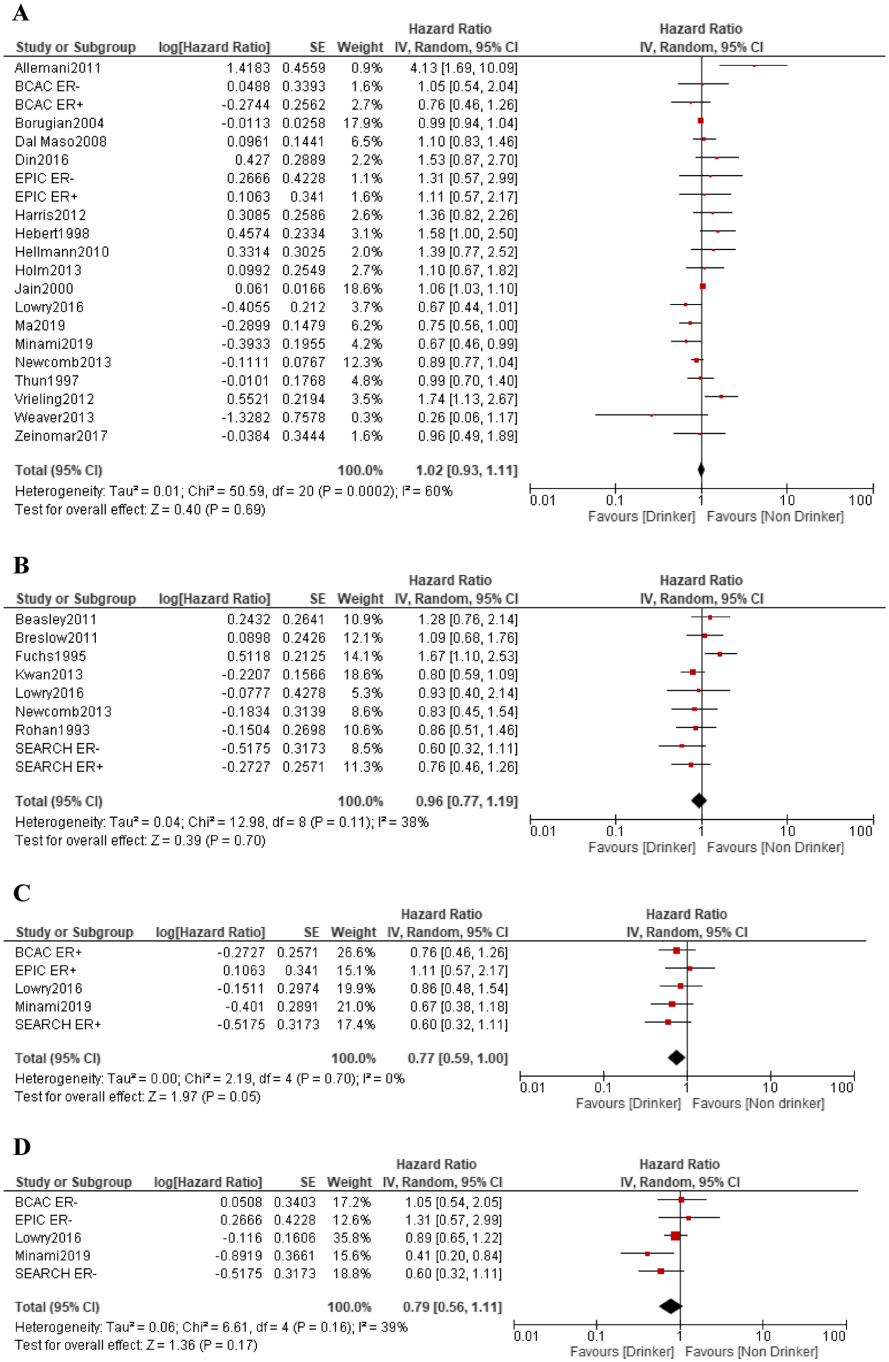
Forest plots of the hazard ratio for alcohol intake and breast cancer-related morality. **A** Pre-diagnosis; **B** post-diagnosis; **C** ER-positive cases; **D** ER-negative cases

In the meta-analysis of 21 studies, the RR for pre-diagnosis alcohol intake was 1.02 (95% CI 0.93–1.11, p = 0.69), showing high heterogeneity in the effect (I^2^ = 60%), but not significant. In nine studies, the RR for post-diagnosis alcohol intake was 0.96 (95% CI 0.77–1.19, p = 0.70), showing slight heterogeneity in the effect (I^2^ = 38%), but not significant (Fig. 3A, B).

In addition, five studies of estrogen receptor (ER) expression status were reported in relation to breast cancer-related death [14, 19, 27], three studies of pre-diagnosis alcohol intake [14, 19] and two studies of post-diagnosis alcohol intake [19, 27]. In the meta-analysis, the RR for the ER-positive cases was 0.77 (95% CI 0.59–1.00, p = 0.05), showing no heterogeneity in the effect (I^2^ = 0%), and the risk was slightly reduced (Fig. 3C). The RR for ER-negative cases was 0.79 (95% CI 0.56–1.11, p = 0.17), showing slight heterogeneity (I^2^ = 39%), but not significant (Fig. 3D).

### Association between alcohol consumption and all-cause mortality

There were 18 pre-diagnosis studies [5, 8, 11, 14–16, 18–21, 26–28, 33, 34] and 8 post-diagnosis studies [12, 13, 16, 19, 29, 30] of alcohol consumption and all-cause mortality in breast cancer patients. For pre-diagnosis alcohol intake, decreased risk was observed in three studies [11, 19, 33]. For post-diagnosis alcohol intake, increased risk was observed in one study [30], with decreased risk in three studies [12, 14, 19].

**Fig. 4:**
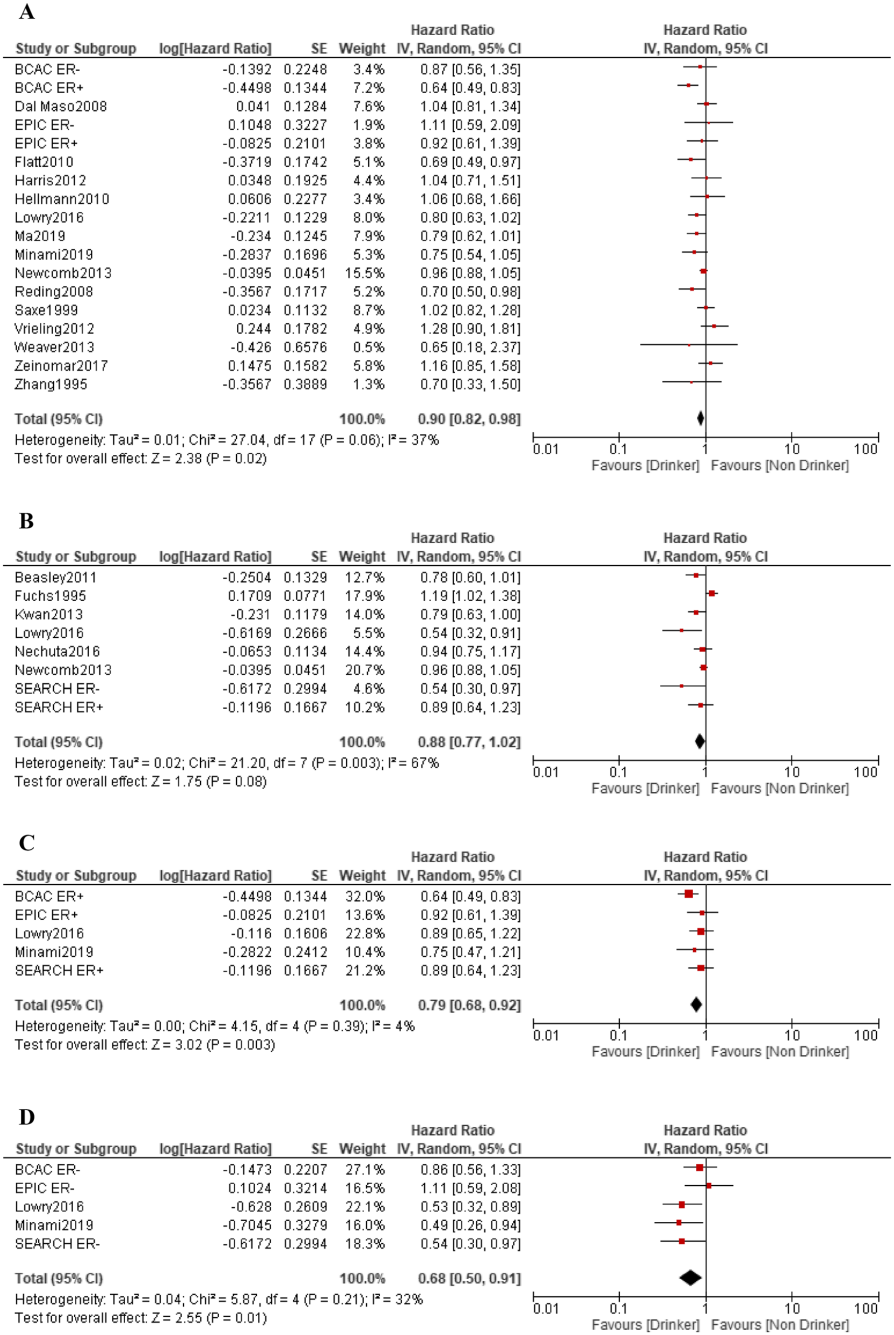
Forest plots of the hazard ratio for alcohol intake and all-cause mortality. **A** Pre-diagnosis; **B** post-diagnosis; **C** ER-positive cases; **D** ER-negative cases

In the meta-analysis of 18 studies, the RR for pre-diagnosis alcohol intake was 0.90 (95% CI 0.82–0.98, p = 0.02), showing slight heterogeneity in the effect (I^2^ = 37%), and the risk was significantly reduced (Fig. 4A). The meta-analysis of seven studies showed that the RR for post-diagnosis alcohol intake was 0.88 (95% CI 0.77–1.02, p = 0.08), showing high heterogeneity in the effect (I^2^ = 67%), and the risk was not significantly reduced (Fig. 4B).

In addition, five studies of ER expression status in relation to all-cause death were reported. Risk reduction was observed in one ER-positive study [19] and three ER-negative studies [14, 19, 27], with three studies of pre-diagnosis alcohol intake [14, 19] and two studies of post-diagnosis alcohol intake [19, 27]. In the meta-analysis, the RR for ER-positive cases was 0.79 (95% CI 0.68–0.92, p = 0.003), showing no heterogeneity in the effect (I^2^ = 4%), and the risk was significantly reduced (Fig. 4c).

The RR for ER-negative cases was 0.68 (95% CI 0.50–0.91, p = 0.01), showing slight heterogeneity (I^2^ = 32%), and the risk was significantly reduced (Fig. 4d).

### Association between alcohol consumption and cardiovascular disease mortality

**Fig. 5:**
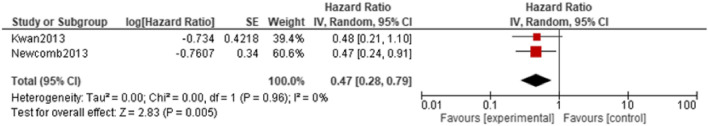
Forest plots of the hazard ratio for alcohol intake and cardiovascular disease mortality

There were two post-diagnosis studies [12, 16] of alcohol consumption and cardiovascular disease mortality in breast cancer patients. For post-diagnosis alcohol intake, decreased risk was observed in one study [16]. In two studies [12, 16], the RR for breast cancer patients was 0.47 (95% CI 0.28–0.79, p = 0.005), showing no heterogeneity in the effect (I^2^ = 0%), and the risk was significantly reduced (Fig. 5).

## Discussion

Alcohol consumption has been reported to increase breast cancer incidence in healthy individuals [35]. However, it is unclear whether alcohol consumption affects breast cancer recurrence, breast cancer-related death, or all-cause death in breast cancer patients. Therefore, a systematic review and meta-analysis of epidemiological studies in breast cancer patients was performed. There were no significant associations between alcohol consumption and breast cancer recurrence [4–13] and breast cancer-related mortality [4, 8, 9, 12, 14–32]. There was high heterogeneity between pre-diagnosis alcohol consumption and breast cancer recurrence and breast cancer-related mortality. This may be due to the insufficient follow-up period in several cases [6, 8, 10, 18, 34] and the variability in the number of events (breast cancer recurrence: 28–1487 cases; breast cancer-related mortality: 26–824 cases) as shown in Table 1. On the other hand, a significant inverse association between pre-diagnosis alcohol consumption and all-cause morality [5, 8, 10, 14–16, 18–21, 26–28, 33, 34] and a significant inverse association between post-diagnosis alcohol consumption and cardiovascular disease mortality were found [12, 16].

Low to moderate amounts of weekly or daily alcohol consumption may be beneficial to cardiovascular health [36, 37]. The reduction in all-cause mortality associated with alcohol consumption after the diagnosis of breast cancer was thought to be related to decreased cardiovascular disease mortality. However, the relationship between alcohol consumption and cardiovascular disease mortality in patients with cardiovascular disease appears to be biphasic and have a J-shaped association [38].

Population level recommendations for upper limits of alcohol consumption exist in many nations as part of public health strategies aimed at reducing adverse health effects. These recommendations vary from country to country. For example, the United States Department of Agriculture recommends women consume not more than 1 standard drink per day (where 1 drink = 14 g of alcohol) [39].

The Ministry of Health, Labour and Welfare of Japan recommends consuming not more than 20 g of alcohol per day. However, this evidence is based on Tsugane’s study [40] and is the result for men. For women, smaller amounts than for men are considered appropriate, because women are smaller than men and are thought to break down alcohol more slowly. Indeed, an increased relative risk of all-cause mortality from alcohol drinking for men is found at 6 or more drinks, whereas for women it is found at 2.0–2.9 drinks [41].

Regarding ER status, we examined it in 5 studies [14, 19, 27]. Regarding the association between alcohol consumption and breast cancer-related death, the HR for ER-positive cases was 0.77 (95% CI 0.59–1.00, p = 0.05), and the HR for ER-negative cases was 0.79 (95% CI 0.56–1.11, p = 0.17). Regarding the association between alcohol consumption and all-cause death, the HR for ER-positive cases was 0.79 (95% CI 0.68–0.92, p = 0.002), the HR for ER-negative cases was 0.68 (95% CI 0.50–0.91, p = 0.01), and the risk was significantly reduced. There was no clear significant difference in risk associated with ER status.

There are several limitations of this systematic review. First, study selection involved filtering for English and Japanese language articles, potentially resulting in the exclusion of relevant, non-English publications. Second, in some studies, analyses were either performed without adjusting for any confounding factors, or analyses did not account for the factors considered most important in regard to the outcome of interest, such as age, BMI, and stage of the initial breast cancer.

Third, since alcohol consumption status is mainly based on questionnaire surveys, the exact amount and frequency of alcohol consumption are not known. In addition, there is the possibility of underreporting.

The present findings led us to create the recommendation for the clinical question in the Japanese Breast Cancer Society Clinical Practice Guideline Committee, 2022 edition, as follows: Whether pre-diagnosis or post-diagnosis, alcohol consumption has a substantial effect on risk and is unlikely to increase the risk of breast cancer recurrence and death from breast cancer.

However, excessive alcohol consumption increases the relative risk of all-cause mortality, and alcohol consumption should be limited to a moderate level as defined by each country.
